# Retrospective analysis of US veterans with inclusion body myositis: initial findings from the Veterans Affairs Corporate Data Warehouse

**DOI:** 10.1186/s40779-025-00592-5

**Published:** 2025-01-28

**Authors:** Vladimir M. Liarski

**Affiliations:** 1 Department of Medicine, Medicine Service Line-Rheumatology, VA Pittsburgh Health Care System, University Drive Campus, Pittsburgh, PA 15240 USA; 2 Department of Medicine, Division of Rheumatology and Clinical Immunology & Department of Immunology, University of Pittsburgh, Pittsburgh, PA 15261 USA

**Keywords:** Idiopathic inflammatory myopathy, Inclusion body myositis (IBM), Interstitial lung disease, Veterans

Dear Editor,

Inclusion body myositis (IBM) is the most common idiopathic inflammatory myopathy in adults over 50 years old [1, 2]. There are no current Food and Drug Administration (FDA)-approved therapies and many unanswered questions regarding disease pathogenesis, course, and outcomes. We leveraged big data resources to conduct a retrospective analysis of IBM patient mortality among United States veterans.

Data were extracted from the Veterans Affairs (VA) Corporate Data Warehouse (CDW) (federal register: 79 FR 4377). Adults over age 18 with an outpatient International Classification of Disease (ICD) 9 or 10 visit code or problem list entry for IBM between January 1, 2011 to December 31, 2021, with a minimum of 2 visits with Neurology or Rheumatology a minimum of 30 d apart were included. Patients with other inflammatory myopathies or autoimmune diseases were excluded. Date of first ICD code or problem list entry was inferred to be date of IBM diagnosis. Diagnoses of smoking and diabetes mellitus or cancer were similarly identified. Patients were matched to controls using the “ccoptimalmatch” R package based on age, sex, race, ethnicity with a zero difference for age and follow-up period enforced [3]. Current Procedural Terminology codes identified patient computerized tomography (CT) imaging studies of the chest/thorax, which were tokenized using “tidytext” and “tokenizers” R packages. Lung involvement was denoted as follows: interstitial lung disease (ILD)—mention of “interstitial lung disease” or ground glass opacities; bronchiectasis—mention of “bronchiectasis” and/or mucus plugging; fibrosis—mention of “fibrosis”, including “honeycombing” or “scarring” changes. We excluded manifestations due to infection, malignancy, or other processes as interpreted by the reading radiologist as well as atelectasis changes and minor lung nodules. The primary outcome was mortality from any cause with a censor date of December 10, 2023. This study was granted informed consent exemption status by the Pittsburgh VA Institutional Review Board Committee based on Revised Common Rule/2018 (Project #1707389–1).

A total of 732 IBM patients and 1215 matched patient controls met study criteria (Additional file 1: Table S1). The follow-up period was (5.6 ± 3.4) years, and 60.9% (446/732) of patients were managed by Neurology, 16.0% (117/732) were treated by Rheumatology, and 23.1% (169/732) were co-managed. Veteran service periods ranged from World War II to the Persian Gulf War with the majority—67.9% (497)—serving during the Vietnam-Era. There was no difference in service periods between IBM and patient controls. IBM veterans were predominantly male (96.7%), White (70.2%), and of non-Hispanic or -Latino ethnicity (89.2%). The mean age of patients was (54.4 ± 8.5) years and mean age at IBM diagnosis was (48.4 ± 8.7) years. Black patients made up 22.1% of IBM veterans and 7.7% identified as Other or did not provide race. Compared with an unpublished analysis performed by our group of 6,789,284 active individuals in the CDW, our cohort contained fewer women (3.3% vs. 9.4%, *P* < 0.0001) and more Black patients (22.1% vs. 17.3%, *P* = 0.0006). The mean maximum creatine phosphokinase level of IBM patients was (925.0 ± 1892.1) U/L. The 5 most common diagnoses among control patients—in order of frequency—were tobacco use (12.2%, 148/1215), primary hypertension (6.1%, 74/1215), gastroesophageal reflux (3.0%, 36/1215), hyperlipidemia (2.0%, 24/1215), and contact or exposure to other hazardous substance (2.0%, 24/1215). IBM patients were more likely to have a diagnosis of diabetes mellitus (49.5% vs. 6.6%, *P* < 0.001) and cancer (38.4% vs. 2.8%, *P* < 0.001), and be current or prior smokers (23.2% vs. 15.8%, *P* < 0.001). The most common malignancies seen in IBM patients were prostate, skin, renal, bladder, and colon cancers, and lymphoma. There were no cases of T cell leukemia as previously reported [4].

Testing for autoantibodies against U1 ribonucleoprotein (U1RNP), the 52 kD isoform of Sjogren Syndrome antigen A (SSA/Ro), and cytosolic 5’-nucleotidase (NT5c1A) in IBM was insufficient for analysis. There were no group differences in exposures to Agent Orange, ionizing radiation, Southwest Asia conditions, military sexual trauma, combat, shipboard hazard defense, and history of head and neck cancer. 134 IBM patients (18.3%) had CT studies in our database compared with 273 (22.5%) of matched controls. Both exhibited similar prevalence of lung scarring/fibrosis (11.9% vs. 16.5%, *P* = 0.290) while IBM patients were more likely to display bronchiectasis (57.5% vs. 15.4%, *P* < 0.001) and ILD (61.9% vs. 48.4%, *P* < 0.001). This difference could not be explained solely by smoking status (23.2% vs. 15.8%, *P* < 0.001) (Additional file 1: Table S1).

Kaplan–Meier survival analysis comparing mortality from any cause between IBM patients and matched controls revealed strikingly worse mean survival among the former [mean 10% mortality for IBM 2.2 years (95%CI 1.8 –2.6) vs. 15.0 years (95%CI 14.0–16.0) for matched controls, *P* < 0.001; Fig. 1]. Univariate and multivariable Cox proportional hazard regressions showed that a diagnosis of IBM had the highest hazard ratio (*HR*) (*HR* = 22.64, 95%CI 17.56–29.20,* P* < 0.001 and *HR* = 18.07, 95% CI 8.54–38.22, *P* < 0.001 for univariate and multivariable analyses, respectively). Age, sex, race, ethnicity, presence of diabetes mellitus, cancer, and imaging evidence of ILD or bronchiectasis were significant in univariate regressions (Additional file 1: Table S2).

**Fig. 1:**
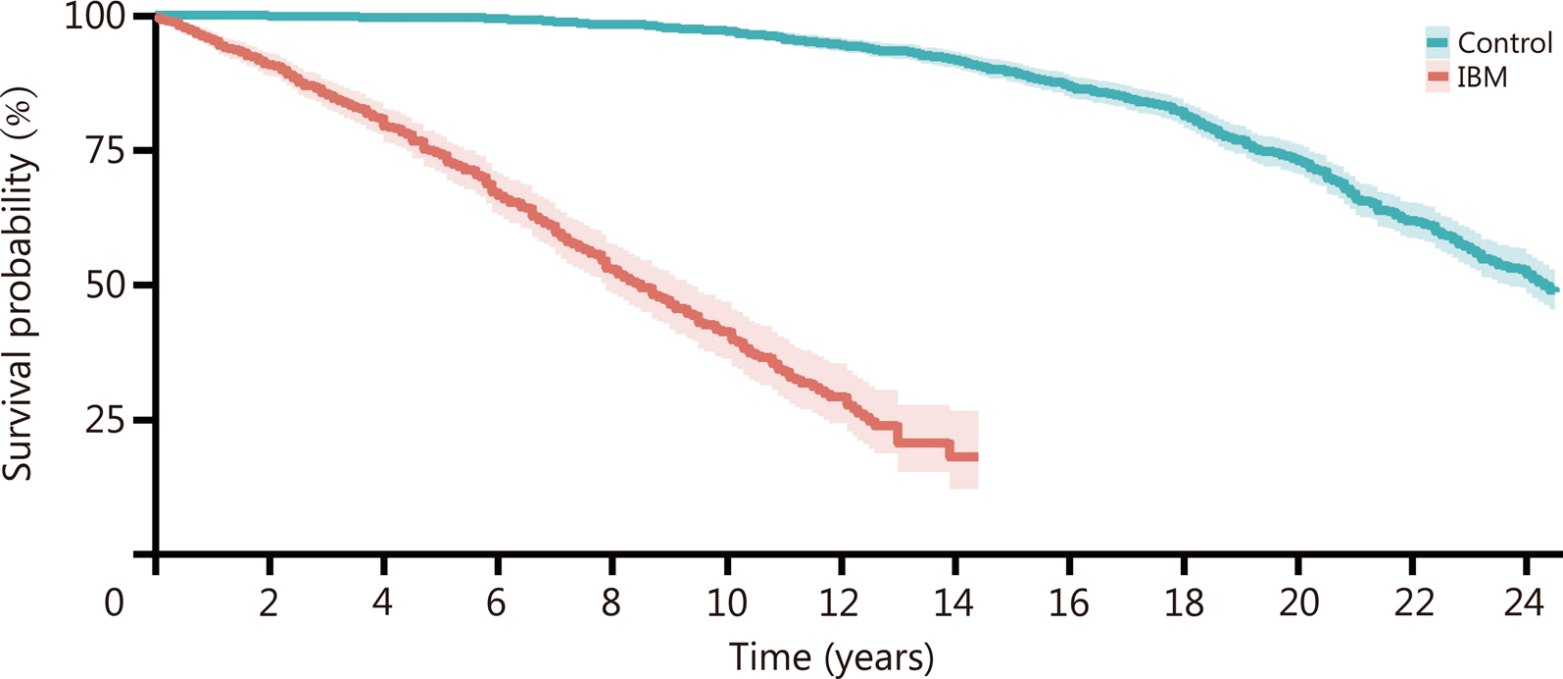
Kaplan–Meier survival plot for inclusion body myositis (IBM) as compared with matched control patients

This is the study of IBM in a veteran population and one of the most racially diverse IBM cohorts examined. Black patients with IBM were overrepresented and all IBM patients exhibited drastically decreased survival (mean 8.5 years) compared with controls. As there are no current treatments for IBM, we do not suspect that potential delays in diagnosis would have influenced our analysis, especially given its age-matched design. Two prior civilian cohorts reported a similar observation: a Dutch analysis reported 15 of 64 (24%) patients alive after 12 years [5], whereas a study from the Mayo clinic found a 10-year survival rate of 36% [4]. It will be important in future work to determine if this mortality is linked to causes or processes unique to IBM and to explore links between IBM and lung disease.

## Supplementary Information

**Additional file 1. Table S1** Comparison of clinical characteristics of inclusion body myositis (IBM) patients with controls. **Table S2** Cox proportional hazard analysis: overall survival of inclusion body myositis (IBM) vs. matched control patients.

## Data Availability

The data used in this study is maintained by the VA CDW and VINCI, is not property of the authors, and is not publicly available. Thus, we are unable to share it with others. Those wishing to access raw data may do so by following the policies laid out by their respective owners.

## Abbreviations

IBM: Inclusion body myositis
CDW: Corporate Data Warehouse
ILD: Interstitial lung disease
CT: Computerized tomography
