# Supplementary material for: Retrospective analysis of US veterans with inclusion body myositis: initial findings from the Veterans Affairs Corporate Data Warehouse
Source: Mil Med Res. 2025 Jan 28;12:6. doi: 10.1186/s40779-025-00592-5 (PMC11773961; doi:10.1186/s40779-025-00592-5)
Supplement: Supplementary file 1 — Additional file 1. Table S1 Comparison of clinical characteristics of inclusion body myositis (IBM) patients with controls. Table S2 Cox proportional hazard analysis: overall survival of inclusion body myositis (IBM) vs. matched control patients. [file 40779_2025_592_MOESM1_ESM.pdf]

Table S1 Comparison of clinical characteristics of inclusion body myositis (IBM) patients with controls

Item	Control (*n* = 1215)	IBM (*n* = 732)	*P*-value
Age (year, mean ± SD)	54.2 ± 8.6	54.4 ± 8.5	0.606
Age at IBM diagnosis (year, mean ± SD)	-	48.4 ± 8.7	NA
Follow-up period (year, mean ± SD)	18.7 ± 5.0	5.6 ± 3.4	< 0.001
Status at end of follow-up period [*n*(%)]			< 0.001
Alive	794 (65.3)	416 (56.8)	
Deceased or censored	421 (34.7)	316 (43.2)	
Sex [*n*(%)]			0.786
Male	1171 (96.4)	708 (96.7)	
Female	44 (3.6)	24 (3.3)	
Race [*n*(%)]			0.772
White	862 (70.9)	514 (70.2)	
Black	227 (18.7)	162 (22.1)	
Other/not provided	126 (10.4)	56 (7.7)	
Ethnicity [*n*(%)]			0.931
Non-Hispanic or -Latino	1081 (89.0)	653 (89.2)	
Hispanic or Latino or other	134 (11.0)	79 (10.8)	
Creatine phosphokinase (U/L, mean ± SD)	-	925.0 ± 1892.1	NA
Smoking status [*n*(%)]			< 0.001
Never	1023 (84.2)	562 (76.8)	
Current or prior	192 (15.8)	170 (23.2)	
Diabetes mellitus [*n*(%)]			< 0.001
Current or prior history	80 (6.6)	362 (49.5)	
Never	1135 (93.4)	370 (50.5)	
Cancer [*n*(%)]			< 0.001
Current or prior history	34 (2.8)	281 (38.4)	
Never	1181 (97.2)	451 (61.6)	
Chest CT [*n*(%)]			0.030
Performed/available	273 (22.5)	134 (18.3)	
Missing/not performed	942 (77.5)	598 (81.7)	

Chest CT findings [*n*(%)]			
ILD			< 0.001
Present	132 (48.4)	83 (61.9)	
Absent	141 (51.6)	51 (38.1)	
Bronchiectasis			< 0.001
Present	42 (15.4)	77 (57.5)	
Absent	231 (84.6)	57 (42.5)	
Fibrosis			0.290
Present	45 (16.5)	16 (11.9)	
Absent	228 (83.5)	118 (88.1)	
Veteran factor [*n*(%)]			
Agent orange			0.109
Present	224 (18.5)	162 (22.2)	
Absent	597 (49.2)	371 (50.7)	
Ionizing radiation			0.675
Present	1 (0.1)	0 (0)	
Absent	167 (13.8)	108 (14.8)	
Southwest Asia conditions			0.806
Present	20 (1.7)	8 (1.2)	
Absent	235 (19.4)	145 (19.9)	
Military sexual trauma			0.266
Present	51 (4.2)	19 (2.6)	
Absent	258 (21.3)	142 (19.4)	
Head neck cancer			0.849
Present	2 (0.2)	1 (0.1)	
Absent	1043 (85.9)	623 (85.2)	
Combat			0.937
Present	43 (3.6)	27 (3.8)	
Absent	229 (18.9)	143 (19.6)	
Shipboard hazard			0.860
Present	17 (1.4)	13 (1.9)	

Absent	239 (19.7)	148 (20.3)	
Veteran service period [*n*(%)]			0.432
World War II	14 (1.2)	6 (0.8)	
Pre-Korean	1 (0.1)	2 (0.3)	
Korean	73 (6.0)	45 (6.1)	
Post-Korean	28 (2.3)	28 (3.8)	
Vietnam-Era	848 (69.8)	497 (67.9)	
Post-Vietnam	118 (9.7)	65 (8.9)	
Persian Gulf War	131 (10.8)	87 (11.9)	
Other/none	1 (0.1)	2 (0.3)	
Non-Veterans	1 (0.1)	0	

P-value represents *t* test for continuous variables and Pearson's χ^2 for categorical variables, $\alpha = 0.05$. All numbers may not add up to 100% due to missing or NULL data. *NA* not available, *ILD* interstitial lung disease, "-" indicates no data

Table S2 Cox proportional hazard analysis: overall survival of inclusion body myositis (IBM) vs. matched control patients

**Factors**	**Univariate analysis**		**Multivariable analysis**	
	***HR* (95%CI)**	***P*-value**	***HR* (95%CI)**	***P*-value**
Disease				
Control	Ref.		Ref.	
IBM	22.64 (17.56 – 29.20)	< 0.001	18.07 (8.54 – 38.22)	< 0.001
Age	1.02 (1.01 – 1.03)	< 0.001	1.00 (0.98 – 1.02)	0.743
Sex				
Male	Ref.		Ref.	
Female	0.29 (0.10 – 0.57)	< 0.001	0.66 (0.24 – 1.81)	0.423
Race				
White	Ref.		Ref.	
Black or other	0.80 (0.68 – 0.95)	0.009	0.94 (0.67 – 1.31)	0.697
Ethnicity				
Non-Hispanic or -Latino	Ref.		Ref.	
Hispanic or Latino or other	4.50 (3.83 – 5.29)	< 0.001	1.19 (0.71 – 2.00)	0.509
Smoking				
Never	Ref.		Ref.	
Current or prior history	1.09 (0.90 – 1.31)	0.380	0.83 (0.55 – 1.23)	0.343
Diabetes mellitus				
Never	Ref.		Ref.	
Current or prior history	3.17 (2.39 – 3.75)	< 0.001	1.20 (0.77 – 1.86)	0.414
Cancer				
Never	Ref.		Ref.	
Current or prior history	3.78 (3.13 – 4.57)	< 0.001	1.20 (0.74 – 1.92)	0.458
ILD on CT				
Absent	Ref.		Ref.	
Present	1.39 (1.02 – 1.88)	0.035	1.39 (0.99 – 1.69)	0.058
Bronchiectasis on CT				
Absent	Ref.		Ref.	

Present	1.58 (1.13 – 2.21)	0.008	0.66 (0.43 – 1.02)	0.060
Fibrosis on CT				
Absent	Ref.		Ref.	
Present	0.99 (0.65 – 1.52)	0.974	1.21 (0.77 – 1.90)	0.396

Multivariable model is adjusted for age, sex, race, ethnicity, smoking status, presence of diabetes mellitus, history of cancer, and presence of interstitial lung disease (ILD), bronchiectasis, or fibrosis on available computerized tomography (CT) imaging
